# Effects of probiotics and amprolium on performance, lesion scores, oocyst shedding, and histopathological changes in *Eimeria tenella*-infected broiler chickens

**DOI:** 10.14202/vetworld.2025.1400-1410

**Published:** 2025-06-06

**Authors:** Thanyakorn Chalalai, Watcharapon Promsut, Kannika Hinkhao, Tirocha Hengphrathani, Kamonporn Sangsakul, Nopparat Bhavabhutanon, Tippayaporn Nonkookhetkhong

**Affiliations:** 1 Stress and Oxidative Stress in Animals Research Unit, Faculty of Veterinary Sciences, Mahasarakham University, Maha Sarakham, Thailand; 2 Faculty of Veterinary Sciences, Mahasarakham University, Maha Sarakham, Thailand; 3 Inspired Nutrient Company Limited, Pathum Thani, Thailand

**Keywords:** amprolium, broilers, coccidiosis, *Eimeria tenella*, intestinal lesions, performance, probiotics

## Abstract

**Background and Aim:**

Coccidiosis caused by *Eimeria tenella* significantly impairs poultry health and productivity, prompting the search for alternative or complementary therapies to conventional coccidiostats. This study investigates the prophylactic and therapeutic efficacy of a probiotic complex of *Lactobacillus*, *Bifidobacteria*, *Enterococcus*, and *Streptococcus*, alone or in combination with amprolium, against *E. tenella* infection in broiler chickens.

**Materials and Methods:**

A total of 90 broiler chickens were randomly allocated into six experimental groups (n = 15/group). Group 1 served as the uninfected control, while Group 2 comprised infected but untreated controls. Group 3 received probiotics at a concentration of 1 mg/mL, and Group 4 was treated with amprolium at a dosage of 20 mg/kg. Group 5 was administered a combination of probiotics (1 mg/mL) and amprolium (20 mg/kg), whereas Group 6 received prophylactic treatment with probiotics (1 mg/mL). All groups except the uninfected control were challenged orally with 2 × 10^4^ sporula-ted *E. tenella* oocysts. Growth performance was monitored on days 15, 21, and 28. Lesion scoring, oocyst shedding, and histopathological examinations were conducted on day 28. An *in vitro* sporulation assay evaluated the inhibitory potential of treatments on oocyst development.

**Results:**

*In vitro*, the probiotic-amprolium combination significantly reduced oocyst sporulation rates (5.86%). *In vivo*, amprolium and prophylactic probiotics significantly improved body weight gain and feed intake (p < 0.05) and reduced oocyst shedding. Lesion severity and parasite stage counts were significantly lower in the amprolium group; however, the combination group exhibited unexpectedly higher lesion scores. Mortality was highest in the amprolium and untreated groups (20%) but absent in the combination group, suggesting differential immunomodulatory effects. Histopathological analysis confirmed reduced intestinal damage in groups treated with amprolium or prophylactic probiotics.

**Conclusion:**

Amprolium monotherapy and prophylactic probiotic supplementation were effective in mitigating *E. tenella*-induced pathology and improving broiler performance. Probiotics alone provided moderate benefits, while their post-infection therapeutic use or co-administration with amprolium did not yield superior results. These findings underscore the prophylactic value of probiotics and warrant further studies to optimize combination regimens under field conditions.

## INTRODUCTION

Coccidiosis remains a disease of global importance in poultry production, severely affecting animal health and economic sustainability. Protozoan parasites of the phylum Apicomplexa, particularly those of the genus *Eimeria*, such as *Eimeria tenella*, frequently cause severe pathological conditions in poultry. These parasites cause extensive intestinal damage, leading to bloody diarrhea, substantial morbidity and mortality, weight loss, reduced feed intake, and, in severe cases, death [1, 2].

The control of coccidiosis in poultry primarily relies on anticoccidial drugs; amprolium, a widely used coccidiostat, acts by competitively inhibiting thiamine absorption [3]. However, continuous usage of anticoccidial drugs has led to the emergence of drug-resistant *Eimeria* strains, raising significant concerns and prompting regulatory restrictions [4]. The widespread presence of resistant pathogens and the high prevalence of virulence and resistance genes in pathogens isolated from poultry and farm workers highlight the zoonotic risk of resistant strains and underscore serious implications for global food safety [5–7]. Isolates of *E. tenella* exhibit severe resistance to salinomycin, diclazuril, and nicarbazin and moderate resistance to amprolium and clopidol [8, 9]. In response to these challenges, alter-native strategies for controlling coccidiosis have been extensively explored. Such alternatives include the use of probiotics, prebiotics, and the combination of anticoccidial drugs with vitamins, minerals, or probiotics to enhance their efficacy against *Eimeria* species [10–12]. Further, public health issues arising from antibiotic residues in poultry products require non-chemical strategies that are urgently needed in poultry farming [13, 14]. Probiotics are non-pathogenic microorganisms that, when administered in sufficient quantities, confer health benefits by maintaining microbial balance, improving digestive function, and promoting overall host health [15]. Common probiotic strains used in poultry include species of *Bifidobacterium*, *Lactococcus*, *Lactobacillus*, *Bacillus*, and *Streptococcus* [16]. The use of probiotics for coccidiosis control offers multiple benefits, including immunomodulation and inhibition of pathogen growth. Probiotics enhance both cell-mediated and humoral immune responses, upregulate Toll-like receptor expression, stimulate cytokine and immun-oglobulin production [17, 18], and promote competitive exclu-sion, thereby reducing the colonization of pathogenic microorganisms [19]. Probiotic supplementation has been demonstrated to mitigate diarrhea and watery stools, attenuate the severity of intestinal lesions, and reduce oocyst shedding following *E. tenella* challenge [20]. In addition, probiotics may alter enterocyte receptor expression, thereby impairing or preventing the penetration of sporozoites and merozoites into host enterocytes [4]. Studies on the combined effects of probiotics and amprolium, as well as comparisons of their use for both prophylactic and therapeutic purposes, are underexplored.

Despite the recognized efficacy of both amprolium and probiotics in mitigating *E. tenella* infections, existing literature predominantly focuses on their individual therapeutic effects rather than their comparative or synergistic potential. Previous studies have highlighted the preventive benefits of probiotics and the anticoccidial effects of amprolium; however, few have rigorously examined their combined use or directly contrasted prophylactic versus therapeutic probiotic applications under standardized experimental conditions. Furthermore, *in vitro* evidence supporting their co-administration has not been consistently validated *in vivo*, and the impact of such comb-ination therapy on lesion severity, oocyst shedding, and histopathological changes remains inadequately characterized. There is also a lack of clarity regarding whether timing (prophylactic vs. post-infection adm-inistration) influences the protective efficacy of probiotics. Furthermore, the lack of data linking mortality outcomes and parasite developmental stages to these interventions further limits the translational application of probiotic or drug-based strategies in commercial poultry settings. Hence, an evidence-based comparative evaluation is critical to determine optimal intervention strategies against *E. tenella*, particularly in the context of growing resistance to anticoccidial drugs and increasing demand for non-antibiotic alternatives.

This study aimed to investigate the individual and combined effects of probiotics and amprolium on broiler chickens experimentally infected with *E. tenella*. Specifically, the study aimed to evaluate their impacts on growth performance, feed intake, feed conversion ratio, lesion severity, oocyst shedding, parasite developmental stages, mortality rates, and histopathological changes. A further objective was to compare the efficacy of prophylactic versus therapeutic probiotic administration and to assess whether the concurrent use of probiotics and amprolium offers additive or synergistic benefits. The overarching goal was to identify effective, sustainable, and practical appro-aches for controlling *E. tenella* infections in poultry production systems while addressing the limitations of current coccidiosis management practices.

## MATERIALS AND METHODS

### Ethical approval

All experimental procedures were approved by the Institutional Animal Care and Use Committee of Mahasarakham University, Thailand (Approval No.: IACUC-MSU-2/2024). The study followed the ARRIVE guidelines for *in vivo* experiment.

### Study period and location

The study was conducted from March to December 2024 at the Faculty of Veterinary Sciences, Mahasarakham University, Thailand.

### Preparation of *E. tenella* oocysts

*E. tenella* oocysts were isolated in 2024 from the cecal contents of naturally infected broiler chickens sourced from farms in Roi Et Province, Thailand. The oocysts were identified morphologically under a light microscope and preserved in 2.5% (w/v) potassium dichromate solution [21]. For sporulation, the oocysts were incubated aerobically at 30°C for 72 h [21]. A total of 2 × 10^4^ sporulated oocysts were orally administered to five 21-day-old commercial broiler chickens. Seven days post-infection, cecal contents were harvested for further experimental use.

### Probiotic supplement

A commercially available, water-soluble multispecies probiotic formulation (Vetafarm Probiotic, Vetafarm Australia Pty Ltd., Wagga Wagga, Australia) was used. The formulation contained *Lactobacillus acid-ophilus*, *Lactobacillus* *delbrueckii* subsp. *bulgaricus*, *Lactobacillus* *plantarum*, *Lactobacillus* *rhamnosus*, *Bifidobacterium* *bifidum*, *Enterococcus faecium*, and *Streptococcus salivarius* subsp. *thermophilus*, with a viable cell count of 180 million colony-forming units/g. It was administered in drinking water at a concentration of 1 mg/mL.

### Amprolium administration

Amprolium (20% amprolium hydrochloride; Advance Pharma Co., Ltd., Samut Prakan, Thailand) was administered through drinking water at a dose of 20 mg/kg.

### *In vitro* sporulation assay

For the *in vitro* sporulation test, 2 × 10^4^ unsporulated oocysts were suspended in 20 mL of different test solutions: (i) probiotics (1 mg/mL), (ii) amprolium (0.6 mg/mL), (iii) a combination of probiotics and amprolium (1 mg/mL + 0.6 mg/mL), and (iv) distilled water as control. Samples were incubated at 25°C–30°C for 72 h. Each treatment was replicated 3 times. The sporulation rate was determined by counting sporulated oocysts using a McMaster counting chamber.

### *In vivo* experiment

#### Animals and experimental design

- Ninety-one-day-old Ross broiler chickens were individually housed in metal cages to prevent reinfection through contact with feces. Birds were randomly assigned to six experimental groups (five birds per group and three replicates per group) and maintained in an open-house system with *ad libitum* access to feed and water. The experimental groups are listed in Table 1. Uninfected and untreated control (UUC)
- Infected and untreated control (IUC)
- Infected and treated with probiotics (Prob)
- Infected and treated with amprolium
- Infected and treated with both probiotics and amprolium
- Infected and pre-treated with probiotics from day 10 to 28 (Prophylactic Prob).

**Table 1:** Experimental groups and descriptions.

Group	Description	Birds/group	Replication
1 (UUC)	Uninfected/untreated control	5	3
2 (IUC)	Infected and untreated controls	5	3
3 (Prob)	Infection and treatment with probiotics	5	3
4 (Amp)	Infected and treated with amprolium	5	3
5 (Prob + Amp)	Infection and treatment with probiotics and amprolium	5	3
6 (Prophylactic Prob)	Infected and pre-treated with probiotics	5	3

Prob=*Lactobacillus*, *Bifidobacteria*, *Enterococcus*, and *Streptococcus,* Prophylactic Prob=Pre-treatment with *Lactobacillus, Bifidobacteria, Enterococcus,* and *Streptococcus*

All groups, except the UUC group, were orally challenged with 2 × 10^4^ sporulated *E. tenella* oocysts at 15 days of age. Treatments were administered from day 20 to 28, except for the prophylactic group. Body weight gain and feed intake were measured on days 15, 21, and 28. Fecal samples were collected on days 22, 25, and 28. Birds were euthanized by cervical dislocation on day 28 for post-mortem sample collection. Randomization was performed using a simple method, and outcome assessors were blinded.

### Oocyst shedding

Fecal samples pooled by group were analyzed to determine oocyst shedding. Samples were mixed with saturated sodium chloride solution and examined using a McMaster counting chamber (0.15 mL per sub-sample) under a light microscope. Results were expressed as oocysts per gram of feces.

### Lesion scoring

Cecal lesion scores were recorded on day 28 post-infection following euthanasia, scoring followed the Johnson and Reid system [22], ranging from 0 (no lesions) to 4 (severe lesions with caseous cores and gross distension).

### Histopathological analysis

Cecal tissues were fixed in 10% neutral-buffered formalin for 24 h, paraffin-embedded, sectioned (4 μm), and stained with hematoxylin and eosin. Histopathological examination was performed under 4×, 10×, and 40× magnifications to assess tissue damage and parasite stages [23]. The number of meronts, gamonts, and oocysts was quantified across 10 villous crypt units in both infected, untreated, and treated groups [24].

### Statistical analysis

Data on growth performance (body weight gain, feed intake, and feed conversion ratio), oocyst counts, and parasite developmental stages were analyzed using one-way analysis of variance followed by Tukey’s *post hoc* test. Lesion scores were analyzed using the Kruskal–Wallis test. Statistical analysis was conducted using IBM SPSS version 29 (IBM Corp., Armonk, NY, USA), with significance set at p < 0.05.

## RESULTS

### *In vitro* sporulation

The sporulation rate of *E. tenella* oocysts was significantly reduced in all treatment groups compared to the control (p < 0.05). Treatments included 1 mg/mL probiotics, 0.6 mg/mL amprolium, and a combination of both. Among these, the combination treatment (1 mg/mL probiotics + 0.6 mg/mL amprolium) resulted in the lowest sporulation rate (Table 2).

**Table 2:** Effect of probiotics and anticoccidial drugs on oocyst sporulation.

Item	Control	Probiotics	Amprolium	Probiotics + Amprolium
Sporulated oocysts	14,200.00 ± 4,133.33^a^	7,400.00 ± 538.86^b^	9,666.66 ± 2,386.37^b^	1,200 ± 307.93^b^
% Sporulated oocysts	69.33	36.13	47.20	5.86

The different superscripts within each group differ significantly (p < 0.05)

### *In vivo* results

#### Growth performance

On day 15, no significant differences were observed among the experimental groups in body weight gain, feed intake, or feed conversion ratio. By day 21, body weight gain and feed intake were significantly higher in the Amprolium, Probiotic + Amprolium, and Prophylactic Probiotic groups compared to the IUC group (p < 0.05). By day 28, all treated groups exhibited significantly greater body weight gain than the IUC group. In addition, the Amprolium group demonstrated significantly higher feed intake compared to the IUC group (p < 0.05). No significant differences in feed conversion ratio were observed on day 21; however, by day 28, the IUC group showed a significantly higher feed conversion ratio compared with all other treated groups (p < 0.05) (Tables 3-5).

**Table 3:** Body weight gain of the experimental chickens.

Group	Body weight gain (g)		

	1–15 days	1–21 days	1–28 days
UUC	385.50 ± 2.50^a^	606.38 ± 7.36^a^	1200.50 ± 173.20^a^
IUC	383.80 ± 2.32^a^	565.13 ± 5.92^b^	963.50 ± 57.00^b^
Prob	383.20 ± 5.45^a^	566.38 ± 2.50^b^	1063.50 ± 129.09^a^
Amp	384.8 ± 5.22^a^	598.40 ± 1.85^c^	1153.50 ± 57.73^a^
Prob + Amp	385.50 ± 4.31^a^	615.55 ± 5.14^a^	1076.50 ± 208.16^a^
Prophylactic Prob	385.30 ± 3.78^a^	584.75 ± 6.07^c^	1099.50 ± 50.00^a^

The different superscripts within each group differ significantly (p < 0.05). UUC=Uninfected and untreated control, IUC=Infected and untreated control, Prob=Infected and treated with probiotics, Amp=Infected and treated with amprolium, Prob + Amp=Infected and treated with both probiotics and amprolium, Prophylactic Prob=Infected and pre-treated with probiotics from day 10 to 28, Prob=*Lactobacillus*, *Bifidobacteria*, *Enterococcus*, and *Streptococcus*, Prophylactic Prob=Pre-treatment with *Lactobacillus*, *Bifidobacteria*, *Enterococcus*, and *Streptococcus*

**Table 4:** Feed intake of experimental chickens.

Group	Feed intake (g)		

	1–15 days	1–21 days	1–28 days
UUC	487.00 ± 28.77^a^	698.50 ± 1.73^a^	1416.75 ± 4.90^a^
IUC	484.00 ± 34.63^a^	655.17 ± 5.58^b^	1486.50 ± 4.35^b^
Prob	470.00 ± 37.84^a^	628.17 ± 48.10^b^	1345.50 ± 6.35^b^
Amp	474.00 ± 34.94^a^	688.58 ± 1.64^a^	1561.00 ± 4.76^c^
Prob + Amp	471.30 ± 33.21^a^	679.00 ± 8.25^a^	1451.25 ± 0.95^b^
Prophylactic Prob	483.60 ± 42.08^a^	698.96 ± 1.20^a^	1425.67 ± 4.00^a^

The different superscripts within each group differ significantly (p < 0.05). UUC=Uninfected and untreated control, IUC=Infected and untreated control, Prob=Infected and treated with probiotics, Amp=Infected and treated with amprolium, Prob + Amp=Infected and treated with both probiotics and amprolium, Prophylactic Prob=Infected and pre-treated with probiotics from day 10 to 28, Prob=*Lactobacillus, Bifidobacteria, Enterococcus*, and *Streptococcus*, Prophylactic Prob=Pre-treatment with Lactobacillus, Bifidobacteria, *Enterococcus*, and *Streptococcus*

**Table 5:** Feed conversion ratios of experimental chickens.

Group	Feed conversion ratio		

	1–15 days	1–21 days	1–28 days
UUC	1.26 ± 0.20^a^	0.87 ± 0.01^a^	1.20 ± 0.17^a^
IUC	1.26 ± 0.11^a^	0.86 ± 0.2^a^	1.55 ± 0.20^b^
Prob	1.23 ± 0.03^a^	0.91 ± 0.08^a^	1.27 ± 0.15^a^
Amp	1.23 ± 0.50^a^	0.87 ± 1.0^a^	1.36 ± 0.13^a^
Prob + Amp	1.22 ± 0.28^a^	0.91 ± 02^a^	1.38 ± 0.28^a^
Prophylactic Prob	1.26 ± 0.08^a^	0.84 ± 0.04^a^	1.30 ± 0.11^a^

The different superscripts within each group differ significantly (p < 0.05). UUC=Uninfected and untreated control, IUC=Infected and untreated control, Prob=Infected and treated with probiotics, Amp=Infected and treated with amprolium, Prob + Amp=Infected and treated with both probiotics and amprolium, Prophylactic Prob=Infected and pre-treated with probiotics from day 10 to 28, Prob=*Lactobacillus, Bifidobacteria, Enterococcus*, and *Streptococcus*, Prophylactic Prob=Pre-treatment with *Lactobacillus, Bifidobacteria, Enterococcus*, and *Streptococcus*

#### Mortality rate

Mortality was recorded on days 23 and 24. The highest mortality rates occurred in the IUC and Amprolium groups (20%), followed by the Probiotic and Prophylactic Probiotic groups (6.67%). No mortality was observed in the Probiotic + Amprolium or UUC groups (Table 6).

**Table 6:** The number and mortality rates of broiler chickens in each group after inoculation.

Group	D23	D24	D25	D26	D28	Mortality rate (%)
UUC	15	15	15	15	15	0
IUC	13	12	12	12	12	20
Prob	14	14	14	14	14	6.67
Amp	13	12	12	12	12	20
Prob + Amp	15	15	15	15	15	0
Prophylactic Prob	15	15	15	14	14	6.67

The different superscripts within each group differ significantly (p < 0.05). UUC=Uninfected and untreated control, IUC=Infected and untreated control, Prob=Infected and treated with probiotics, Amp=Infected and treated with amprolium, Prob + Amp=Infected and treated with both probiotics and amprolium, Prophylactic Prob=Infected and pre-treated with probiotics from day 10 to 28, Prob=*Lactobacillus, Bifidobacteria, Enterococcus,* and *Streptococcus*, Prophylactic Prob=Pre-treatment with *Lactobacillus, Bifidobacteria, Enterococcus*, and *Streptococcus*

#### Oocyst shedding

**Figure 1:**
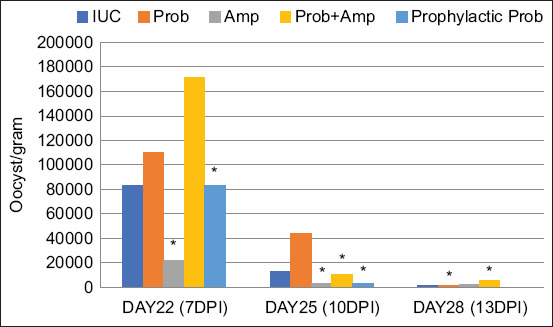
Oocyst shedding patterns of *Eimeria tenella* in chicken feces. *Indicates statistically significant difference (p < 0.05). UUC=Uninfected and untreated control, IUC=Infected and untreated control, Prob=Infected and treated with pro-biotics, Amp=Infected and treated with amprolium, Prob + Amp=Infected and treated with both probiotics and amprolium, Prophylactic Prob=Infected and pre-treated with probiotics from day 10 to 28, DPI=day post infection. Prob=*Lactobacillus, Bifidobacteria, Entero-coccus*, and *Streptococcus*, Prophylactic Prob=*Lactoba-cillus, Bifidobacteria, Enterococcus*, and *Streptococcus*

On 7 days post-infection (day 22), oocyst shed-ding was significantly lower in the Amprolium and Prophylactic Probiotic groups compared to the IUC group (p < 0.05). This trend persisted at 10 days post-infection (day 25), with these two groups continuing to show the lowest oocyst counts, followed by the Probiotic + Amprolium group. By day 28, the Probiotic and Prophylactic Probiotic groups maintained significantly lower oocyst counts than the IUC group (p < 0.05) (Figure 1).

#### Lesion scores

On day 28, lesion scoring revealed significantly lower values in the UUC group compared to all other groups (p < 0.05). The Amprolium-treated group exhibited significantly reduced lesion severity relative to the IUC group (p < 0.05). However, the Probiotic, Probiotic + Amprolium, and Prophylactic Probiotic groups did not differ significantly from the IUC group. Notably, the highest lesion scores were observed in the Probiotic + Amprolium group, even exceeding those of the IUC group (Table 7).

**Table 7:** Lesion scores of experimental chickens.

Group	Lesion scores
UUC	0.00 ± 0.00^a^
IUC	2.00 ± 1.22^b^
Prob	1.00 ± 0.00^b^
Amp	0.40 ± 0.55^ab^
Amp + Prob	2.40 ± 1.51^b^
Prophylactic Prob	1.20 ± 1.10^b^

The different superscripts within each group differ significantly (p < 0.05). UUC=Uninfected and untreated control, IUC=Infected and untreated control, Prob=Infected and treated with probiotics, Amp=Infected and treated with amprolium, Prob + Amp=Infected and treated with both probiotics and amprolium, Prophylactic Prob=Infected and pre-treated with probiotics from day 10 to 28, Prob=*Lactobacillus, Bifidobacteria, Enterococcus*, and *Streptococcus*, Prophylactic Prob=Pre-treatment with *Lactobacillus, Bifidobacteria, Enterococcus*, and *Streptococcus*

#### Histopathological findings

**Figure 2:**
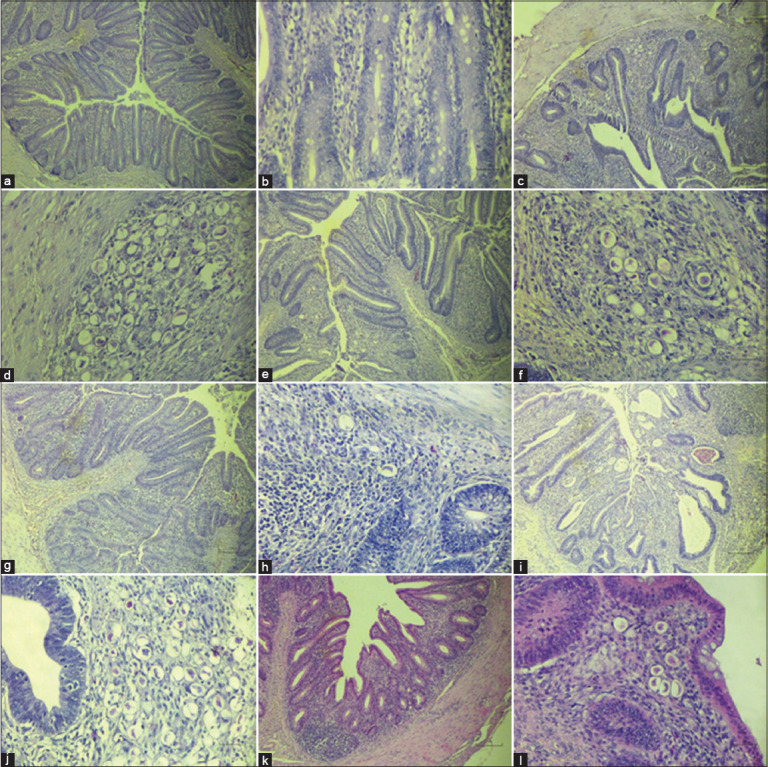
Section of the cecal tissue of experimental chickens. (a and b) UUC group, (c and d) IUC group, (e and f) Prob group, (g and h) Amp group, (i and j) Amp + Prob group, and (k and l) Prophylactic probiotic group. Scale bar: 100 μm. UUC=Uninfected and untreated control, IUC=Infected and untreated control, Prob=Infected and treated with probiotics, Amp=Infected and treated with amprolium, Prob + Amp=Infected and treated with both probiotics and amprolium, Prophylactic Prob=Infected and pre-treated with probiotics from day 10 to 28.

Cecal tissue from the UUC group showed no pathological changes (Figures 2a and b). In contrast, the IUC group displayed severe tissue damage, including villus shortening, crypt swelling, hemorrhage, and a dense presence of developmental stages of *E. tenella* in over 50% of the lamina propria (Figures 2c and d). The Prob group exhibited moderate tissue alterations, characterized by visible oocyst clusters, congestion, and inflammatory infiltration, although less severe than in the IUC group (Figures 2e and f). The Amprolium group demonstrated only mild damage, with <10% oocysts and gametocytes and fewer inflammatory cells (Figures 2g and h). Conversely, the Probiotic + Amprolium group exhi-bited extensive epithelial damage, crypt swelling, desquamation, and parasite clusters affecting over 50% of the tissue, with heavy inflammatory infiltration (Figures 2i and j). In the Prophylactic Probiotic group, moderate lesions were observed with 20% of samples showing oocyst clusters and fewer inflammatory cells (Figures 2k and l).

**Figure 3:**
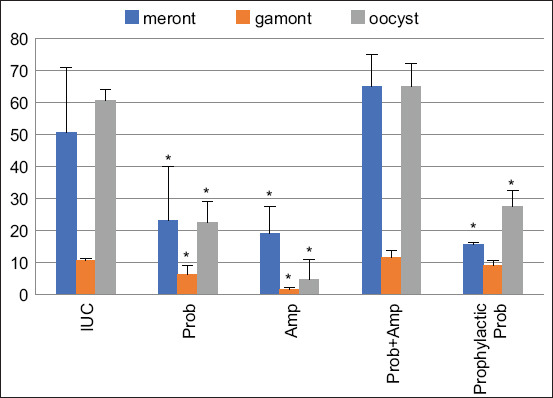
The number of *Eimeria tenella* stages, including meront, gamont, and oocyst in cecal tissue of experimental chickens. *Indicates statistically significant difference (p < 0.05). UUC=Uninfected and untreated control, IUC=Infected and untreated control, Prob=Infected and treated with probiotics, Amp=Infected and treated with amprolium, Prob + Amp=Infected and treated with both probiotics and amprolium, Prophylactic Prob=Infected and pre-treated with probiotics from day 10 to 28. Prob=*Lactobacillus, Bifidobacteria, Enterococcus*, and *Streptococcus*, Prophylactic Prob=*Lactobacillus, Bifido-bacteria, Enterococcus*, and *Streptococcus*

Quantitative assessment revealed that meront, gamont, and oocyst counts were significantly lower in the Probiotic and Amprolium groups compared to the IUC group. The Prophylactic Probiotic group also showed reduced numbers of meronts and oocysts. However, parasite stage counts were unexpectedly higher in the Probiotic + Amprolium group than in the IUC group (Figure 3).

## DISCUSSION

### *In vitro* compatibility of amprolium and probiotics

Previous studies by Nonkookhetkhong and Chalalai [10], Nahed *et al*. [25], and Cai *et al*. [26] have demonstrated that both probiotics and antibiotics exert preventive effects against *Eimeria* spp. infections. This study investigated the individual and combined effects of probiotics and amprolium against *E. tenella* infection, encompassing both prophylactic and therapeutic applications. Our *in vitro* experiments revealed that the combination of amprolium and probiotics was the most effective regimen for inhibiting oocyst sporulation. This finding is consistent with Kant *et al*. [3], who demonstrated that amprolium inhibits thiamine utilization by competitively binding to thiamine receptors, thereby disrupting structural synthesis essential for oocyst development. Moreover, *Lactobacillus* species have been shown to effectively inhibit *E. tenella* in Madin-Darby bovine kidney cell models [27], further supporting the potential benefits of combining probiotics with amprolium. These results suggest that amprolium and probiotics do not exhibit antagonistic interactions *in vitro*.

### Growth performance and mortality outcomes

Probiotic administration, whether prophylactically or therapeutically, significantly improved growth performance in the final week of the experiment, particularly body weight gain and markedly reduced mortality rates. Post-mortem examinations confirmed that mortality was directly caused by *E. tenella* infection. Probiotic supplementation improved body weight gain, feed conversion ratio, and meat quality, reflecting better nutrient absorption and metabolic efficiency. *Bacillus subtilis* has been reported by Gul and Alsayeqh [28], Magnoli *et al*. [29], and Yu *et al*. [30] to improve not only growth performance but also bone integrity, gut morphology, and cecal microbiota composition in broiler chickens. Probiotics enhance intestinal function, optimize microbial composition, maintain microbial balance, reduce pathogenic bacterial load, and increase digestive enzyme activity, thereby promoting overall growth performance [31–33]. In addition, probiotics stimulate both cell-mediated and humoral immune responses and produce lactic acid, which inhibits pathogenic bacterial proliferation, providing substantial immunogenicity and protecting intestinal integrity [20]. Even in the presence of severe intestinal damage induced by *E. tenella*, probiotics have demonstrated the ability to colonize unaffected gut regions, thereby preventing further pathogen invasion [34].

### Oocyst shedding and prophylactic efficacy

Prophylactic probiotic administration was the most effective strategy for reducing oocyst shedding during the later stages of infection. This observation aligns with prior reports by El-Ghany *et al*. [35] and Dalloul *et al*. [36] that prophylactic probiotics moderately improve growth performance and significantly reduce oocyst counts in comparison to anticoccidial drug treatments. Given that *Eimeria* and *Lactobacillus* share intestinal niches, it is plausible that *Lactobacillus* inhibits or reduces *Eimeria* infection through competitive exclusion and modulation of gut microbiota [26, 27]. Prophylactic supplementation contributes to establishing a more stable and beneficial gut microbiota, which is critical for preserving intestinal integrity and function during pathogenic challen-ges [37]. Furthermore, early probiotic administration may prom-ote the production of immunomodulatory substances such as short-chain fatty acids and stimul-ate the innate immune system, offering robust early-stage protection against infection [38]. In contrast, post-infection probiotic supplementation led to higher oocyst shedding compared to prophylactic treatment. This may be due to the fact that once epithelial damage and inflammation have occurred, probiotics are less effective in reducing oocyst output [39]. These results suggest that probiotic supplementation, whether used for prevention, treat-ment, or in combination with anticoccidial drugs, effecti-vely reduces *Eimeria*-associated mortality in broiler chickens.

#### Amprolium effectiveness and associated mortality

Similarly, amprolium treatment significantly improved growth performance and reduced oocyst shedding. This is consistent with the findings of El-Ghany *et al*. [35], who reported that amprolium-treated chickens had lower oocyst counts per gram of feces compared to those treated with probiotics alone. Amprolium treatment was also associated with reduced lesion scores and minimal histopathological alterations, including fewer schizonts, gametocytes, and oocysts, supporting previous findings by Ogwiji *et al*. [34] and Trujillo-Peralta *et al*. [40]. Its mechanism of action involves competitive inhibition of thiamine receptors, disrupting parasite metabolism, inhibiting merozoite development, blocking second-generation meront formation, and partially impairing sexual stages and oocyst sporulation [3, 41]. However, unlike previous findings by El-Ghany *et al*. [35], the present study observed the highest mortality rate in the amprolium-treated group. Severe blood loss caused by *E. tenella*-induced intestinal damage, compounded by impaired nutrient absorption and oxidative stress induced by opportunistic pathogens, is known to be the principal cause of death in untreated infected chickens [1, 42]. Importantly, amprolium lacks intrinsic antibacterial and immunostimulatory effe-cts, which may contribute to gut dysbiosis, facilitate the proliferation of *Clostr-idium perfringens* and *Escherichia coli*, reduce *Lactobacillus* populations, and promote bacterial translocation [26, 43].

#### Ineffectiveness of combined therapy

Unexpectedly, the combination of probiotics and amprolium was ineffective at reducing either oocyst shedding or cecal lesion severity. The combination group exhibited higher lesion scores than any other treatment group, even exceeding those of the IUC group. This finding corroborates previous reports by Ritzi *et al*. [31] and Dalloul and Lillehoj [39] that such combinations do not consistently reduce oocyst shedding or lesion severity *in vivo*. One potential explanation is that lactic acid produced by *Lactobacillus* increases gut acidity, which may influence the chemical stability or solubility of amprolium, despite its general pH stability [44, 45]. Although amprolium is chemically stable across a broad pH range, extreme deviations could potentially alter its molecular structure or bioavailability, reducing its anticoccidial efficacy. In addition, amprolium’s mode of action primarily targets *Eimeria* schizonts and does not confer antibacterial activity. This may allow opportunistic pathogens to proliferate within the intestinal environment, ther-eby exacerbating epithelial damage [46]. These find-ings suggest that the combined use of probiotics and amprolium may not be an effective strategy for coccidiosis control in broiler chickens. Instead, either prophylactic probiotics or amprolium monotherapy may offer more reliable therapeutic outcomes.

### Limitations and practical implications

A key limitation of this study was the absence of gut microbiota analysis, which precluded a detailed understanding of microbial shifts following treatment. From a practical standpoint, probiotics represent a promising intervention for both prevention and miti-gation of *E. tenella* infections. In cases where therapeutic intervention is required post-infection, probiotics may offer an alternative to reduce clinical losses, particularly for farms aiming to limit antimicrobial use. Nonetheless, the efficacy of probiotics remains lower than that of amprolium in terms of parasite suppression and lesion control.

### Future research

Further investigation is warranted to optimize probiotic strain selection, dosing regimens, and timing, particularly when combined with anticoccidial agents. In addition, future studies should incorporate gut microbiota profiling and immune response markers to better understand the interactions between host, microbiota, and pathogen. Field-based evaluations will also be essential for determining the practicality and scalability of combined probiotic–amprolium regimens in commercial poultry systems.

## CONCLUSION

This study demonstrated that both amprolium and probiotics confer protective effects against *E. tenella* infection in broiler chickens, with distinct advantages depending on the treatment strategy. *In vitro*, the combination of amprolium and probiotics significantly inhibited oocyst sporulation, indicating potential compatibility under controlled conditions. *In vivo*, however, prophylactic probiotic administration and amprolium monotherapy proved more effective than their combined application.

Amprolium monotherapy significantly improved growth performance, reduced oocyst shedding, and minimized lesion severity and histopathological alterations. Nevertheless, its association with elevated mortality rates highlights limitations due to the absence of immunomodulatory and antibacterial activity. Probiotics, particularly when administered prophy-lactically, enhan-ced weight gain, reduced mortality, and effectively lowered oocyst excretion. These benefits likely result from improved gut health, imm- une modulation, and competitive exclusion of *Eimeria*.

Contrary to expectations, the co-administration of probiotics and amprolium did not yield synergistic effects. Instead, the combination group exhibited higher lesion scores and parasite burdens, possibly due to pH-related interference or microbial dysbiosis. This finding underscores the importance of assessing pharmacodynamic compatibility when designing combination therapies.

The strengths of this study include its comprehensive assessment of performance metrics, parasitological outcomes, lesion scoring, and histopa-thology. However, a major limitation was the absence of gut microbiota analysis, which would have provided mechanistic insight into microbial shifts and treatment efficacy.

In practical terms, probiotics offer a viable alternative or adjunct to anticoccidials, particularly in antibiotic-restricted production systems. However, their efficacy remains lower than that of amprolium in parasite suppression. Future studies should focus on optimizing the selection of probiotic strains, dosing schedules, and the timing of administration. Moreover, the inclusion of microbiome and immunological analyses, alongside field-based evaluations, will be critical for validating the efficacy and feasibility of integrated coccidiosis control strategies in commercial poultry production.

## DATA AVAILABILITY

All the generated data are included in the manuscript.

## AUTHORS’ CONTRIBUTIONS

TC and TN: Designed and executed the experiment. WP, KH, TH, HS, and NB: Collected the data and performed the experimental work. TC, WP, and TN: Analyzed the data. TC and TN: Drafted and revised the manuscript. All authors have read and approved the final manuscript.
